# MicroRNAs and MAPKs: Evidence of These Molecular Interactions in Alzheimer’s Disease

**DOI:** 10.3390/ijms24054736

**Published:** 2023-03-01

**Authors:** Ivana Raffaele, Serena Silvestro, Emanuela Mazzon

**Affiliations:** IRCCS Centro Neurolesi Bonino Pulejo, Via Provinciale Palermo, Contrada Casazza, 98124 Messina, Italy

**Keywords:** microRNAs, MAPK, Alzheimer’s disease, neurodegeneration

## Abstract

Alzheimer’s disease (AD) is a neurodegenerative disorder known to be the leading cause of dementia worldwide. Many microRNAs (miRNAs) were found deregulated in the brain or blood of AD patients, suggesting a possible key role in different stages of neurodegeneration. In particular, mitogen-activated protein kinases (MAPK) signaling can be impaired by miRNA dysregulation during AD. Indeed, the aberrant MAPK pathway may facilitate the development of amyloid-beta (Aβ) and Tau pathology, oxidative stress, neuroinflammation, and brain cell death. The aim of this review was to describe the molecular interactions between miRNAs and MAPKs during AD pathogenesis by selecting evidence from experimental AD models. Publications ranging from 2010 to 2023 were considered, based on PubMed and Web of Science databases. According to obtained data, several miRNA deregulations may regulate MAPK signaling in different stages of AD and conversely. Moreover, overexpressing or silencing miRNAs involved in MAPK regulation was seen to improve cognitive deficits in AD animal models. In particular, miR-132 is of particular interest due to its neuroprotective functions by inhibiting Aβ and Tau depositions, as well as oxidative stress, through ERK/MAPK1 signaling modulation. However, further investigations are required to confirm and implement these promising results.

## 1. Introduction

Alzheimer’s disease (AD), with more than 50 million people affected worldwide, is the most common form of dementia and poses a major public health burden for patients and their families [1]. This pathology manifests itself with memory loss and behavioral changes up to the loss of daily life activities that force the patient to become completely dependent on family or caregivers. Therefore, AD is a devastating and deadly disease and represents a major challenge for researchers [2]. The causes that induce AD are partially clear. However, genetics, environment, and lifestyle play an important role in AD pathogenesis, and certainly, aging remains the main risk factor. Extracellular deposition of amyloid-beta (Aβ) and neurofibrillary tangles (NFT) produced by hyperphosphorylated Tau protein (p-Tau), together with neuronal loss, are the hallmarks of this pathology [3,4]. The pathogenesis of AD depends on several factors, including apolipoprotein E (*APOE*) genetic variants, the APOE phenotype, and oxidative stress, which can promote damage to both DNA and RNA, including non-coding RNA (ncRNA) [5]. Among ncRNAs, microRNAs (miRNAs) are known to contribute to disease processes in AD [6,7]. Indeed, miRNAs are small ncRNAs of approximately 22 nucleotides that regulate messenger RNA (mRNA) expression, playing a crucial role in different biological processes [8]. The aberrant expression of specific miRNAs, such as miR-34a, miR-125b, and miR-155, has been previously associated with central nervous system (CNS) diseases [9,10,11,12,13]. In this regard, Prendecki et al. 2019 [5] highlighted that plasma levels of miR-107 and miR-650 in AD patients, quantified by quantitative PCR (qPCR), may be related to *APOE* genetic variants and clinical characteristics, including the age of onset and severity of dementia. Age of onset in AD patients, symptom severity, and *APOE* genetic variants may influence the regulation of APOE, miR-107, and miR-650 levels. The strongest relationship between APOE level and miRNA appears in patients with onset at 60–69 years of age and in patients with the *APOE E3*/*E3* genotype. Thus, altered levels of plasma APOE, miR-107, and miR-650 may be a marker of the neurodegenerative process in the course of AD, associated with Aβ metabolism and disordered cell cycle [5]. According to several studies, many other miRNAs have the potential as biomarkers of disease since their deregulation has been found in the serum, plasma, and cerebrospinal fluid (CSF) of AD patients compared to healthy controls [14,15]. However, the effects of miRNA aberrant expression are still not entirely clear. MiRNAs’ dysregulations have been found in different neuropathological processes, including protein aggregation and inflammation [16,17,18]. Thus, several alterations may affect a number of molecular signaling pathways. In this context, recent data have reported that aberrant Mitogen-activated protein kinases (MAPKs) levels might be associated with cognitive dysfunction and could accelerate AD progression [19,20]. According to the literature, MAPKs may represent potential targets for AD. Indeed, their inhibition could prevent Aβ deposition, Tau hyperphosphorylation, neuronal apoptosis, and memory impairment [21]. The modulation of MAPK signaling by miRNA was previously evidenced, especially in cancer [22,23,24,25], but the interplay between miRNAs and MAPKs in neurodegenerative disease remains to be elucidated. Thus, exploring the possible effects of miRNA deregulation on MAPK signaling during AD would be an interesting chance in the diagnostic and therapeutic field. MAPKs are serine and threonine protein kinases expressed in both neuronal and non-neuronal cells of the mature CNS [26]. In response to several external stimuli, such as growth factors, glutamate and hormones, cellular stress, and pathogens [27], MAPKs mediate cell proliferation, differentiation, and survival [28]. Among the different MAPK enzymes, the most studied are extracellular signal-regulated kinases 1 and 2 (ERK1/2), ERK5, c-Jun amino-terminal kinase (JNK) 1 to 3, and p38 MAPK (α, β, γ, and δ) [29]. JNK and p38 MAPK are also known as stress-related protein kinases because they are strongly activated in several disease processes, including AD-associated β-amyloid neurodegeneration [28,30,31,32].

In this review, we provide an overview of evidence that evaluates the molecular interactions between miRNAs and MAPK pathways using in vitro and in vivo experimental AD models. In particular, the pathophysiology of AD will be illustrated, detailing the potential role of miRNAs and MAPKs in this condition. Furthermore, the biogenesis and structure of miRNAs and the role of MAPKs in AD will be mentioned. In order to select the manuscripts, we proceeded to search on PubMed and Web of Science using the following keywords “miRNAs” and “MAPK” or “map kinase” and “or “p38” or “jnk” or “ERK” and “Alzheimer’s disease”; publications ranging from 2010 to 2023 were selected.

## 2. Alzheimer’s Disease

AD is a neurodegenerative disorder characterized by neuron loss and tissue damage, with progressive cognitive impairment [33]. The presence of amyloid plaque and NFT in different regions of the brain are considered the hallmarks of AD, as well as glia activation and endosome enlargement [34].

The extracellular accumulations of Aβ are responsible for triggering a complex of the pathological network that causes neuronal damage [35]. Aβ peptide is generated by the enzymatic proteolysis of the amyloid precursor protein (APP), a protein that physiologically has a key role in brain homeostasis [36]. In healthy subjects, APP is cleaved by an α-secretase and generates the soluble peptide APPα (sAPPα), a molecule involved in neuronal plasticity and survival and protection against cytotoxicity [37,38]. α-secretase-mediated APP processing represents the non-amyloidogenic pathway. However, Aβ peptide is produced following a β- and γ-secretase-mediated amyloidogenic pathway in AD subjects [39]. APP undergoes a first cleavage induced by the enzymatic cleavage of β-secretase 1 (BACE1), which generates the soluble peptide APPβ (sAPP-β) and a fragment consisting of 99 amino acids. sAPP-β is further cleaved by γ-secretase, generating a peptide of 40 amino acids and a peptide of 42, called Aβ_1–40_ and Aβ_1–42_, respectively [40,41]. The latter is more hydrophobic, amyloidogenic, and toxic [42,43]. After releasing in extracellular space, Aβ_1–42_ peptides form the “amyloid plaque” due to their higher propensity to aggregate compared to physiological products [44]. Of note, another key player in AD is Tau, a protein that stabilizes microtubules and promotes vesicular-mediated transport [45]. In physiological conditions, the Tau protein is in perfect balance between phosphorylated and dephosphorylated forms. In patients with AD, hyperphosphorylation leads to the formation of NFT in the cell body of neurons with consequent destabilization and neuronal death [46]. The presence of Aβ and Tau depositions have been associated with synaptic and neuron loss and, as a consequence, with the development of AD symptoms [4]. Therefore, Aβ and Tau interact with each other, promoting pathogenesis and neurodegeneration through different mechanisms that can involve MAPK signaling [47]. Moreover, several pieces of evidence proved the main role of oxidative stress in the early stages of AD by inducing modifications in the cerebral tissue before the formation of Aβ-plaques and NFT [48,49]. The brain is particularly predisposed to oxidative damage due to its high oxygen consumption, high lipid content, and low levels of antioxidant enzymes [48]. Reactive oxygen species (ROS), including peroxide oxygen (H_2_O_2_) and hydroxyl radicals, can induce cell death and senescence. It has been observed that the alteration in miRNA expression levels, induced as a response to ROS, can play a potential role in the pathogenesis of AD. Growing evidence has proven the role of neuroinflammation in the pathogenesis and progression of AD through the activation of microglia [50]. Indeed, neuroinflammation plays an important role in the onset and progression of neurodegeneration and neuronal loss in neurodegenerative diseases [51]. If prolonged over time, the inflammatory response can be deleterious because of the release of toxic substances by chronically activated microglia [52]. Neuroinflammation seems to play a critical role also in the dysregulation of mitochondrial and synaptic processes, which has been strongly correlated with AD pathogenesis [50,53]. However, the specific mechanisms of AD pathology are still not entirely clear. In terms of clinical manifestations, memory deficit is the most common symptom, although other important impairments involve language, visuospatial function, and executive function [44]. The loss of two or more cognitive domains is referred to as dementia [54]. Importantly, at present, there is no cure or treatment that can stop the progression of dementia. Despite the efforts to find potential targets, no medication has ever been approved following a clinical trial. The current drugs, which are acetylcholinesterase inhibitors and N methyl D aspartate receptor antagonists, can only slow the onset of the symptoms [55]. Indeed, AD is estimated to become one of the most devastating diseases of this century, in terms of costs and mortality, since it is the main cause of dementia worldwide [56]. Although AD usually occurs in a sporadic form in people > 65 years of age, a small percentage of patients develop earlier onset associated with mutations in different genes, including *APP*, *PSEN2*, and *PSEN1* (encoding, respectively, amyloid precursor protein, presenilin-1, and presenilin-2) [57]. PSEN1 and PSEN2 are A proteases that regulate the functions of the γ-secretase enzyme, responsible for cutting the amyloid protein. Thus, mutations due to these genes cause the accumulation of Aβ. Instead, late-onset AD is mainly associated with a polymorphism in the *APOE* gene encoding APOE, a protein involved in lipid metabolism [58,59]. In particular, the APOE4 isoform may influence the pathogenesis of AD by promoting the conversion of Aβ into a fibrillar form and its deposition [60].

However, exploring other risk factors and/or biomarkers of AD could be helpful. In the last decade, researchers have highlighted the role of the intestinal microbiota in the pathogenesis of neurological and metabolic disorders [61,62,63]. The microbiota–gut–brain axis is a bidirectional communication system between the CNS and the gastrointestinal (GI) tract [64,65]. Natural bacteria living in the GI are involved in the modulation of immune responses and digestive processes, such as carbohydrate fermentation and vitamin synthesis [66]. On the other hand, intestinal dysbiosis could contribute to different stages of AD pathogenesis through the secretion of neuroactive molecules [67,68,69,70] and harmful metabolites, such as lipopolysaccharide (LPS). LPS is a bacterial endotoxin that triggers the release of pro-inflammatory cytokines and superoxide [71,72]. Furthermore, LPS has been shown to cause increased levels of APP and phosphorylated Tau in a PC12/THP-1 cell model [73]. In particular, gut-derived compounds can increase intestinal permeability, leading to the transport of harmful metabolites across the gut–brain axis to the brain. Thus, the gut microbiome seems to be involved in neurodegenerative diseases, including Parkinson’s disease and AD, but also in neuropsychiatric disorders such as depression and autistic spectrum disorders [69,74,75]. Previous studies demonstrated that the composition and diversity of the gut microbiota are altered in AD patients compared to cognitively normal controls [76]. However, evidence using AD animal models showed positive effects after treatment with antibiotics, probiotics, diet modification, or after fecal microbiota transplantation [63,77]. Thus, modulation of the gut microbiota could be a possible therapeutic and preventive intervention to alleviate symptoms or slow down the progression of AD [78,79,80].

In most cases, the first stages of pathology remain asymptomatic for many years, while the development of the disease depends on several risk factors, such as age and sex. Thus, the diagnosis of AD is complex and requires various analyses, including neurological and physical tests and above all, brain imaging [81]. The most accurate diagnosis is still the post-mortem histological examination, through which the characteristic lesions of AD can be identified [82]. Over recent decades, research has focused on the discovery of biomarkers that may promote both preclinical diagnosis and novel treatments for AD. Early identification of patients with AD could facilitate therapeutic intervention even before cognitive impairment. Indeed, the discovery of new molecular targets during the first stage of neurodegeneration may help to stop the pathogenesis of AD [83,84].

## 3. MiRNAs Biogenesis, Structure, and Function

**Figure 1:**
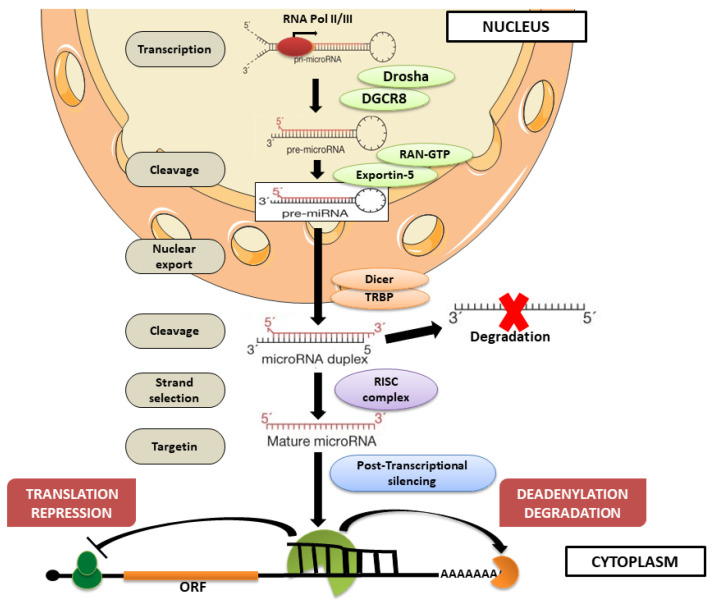
The miRNA biogenesis process. RNA Pol II/III transcribed miRNA genes to form pri-miRNA transcript. Pri-miRNAs are processed by microprocessor complex Drosha–DGCR8 in the nucleus to generate pre-miRNAs. After translocation into the cytoplasm by exportin-5–Ran-GTP, pre-miRNAs are processed by RNase Dicer to form the mature miRNA duplex. Subsequently, one strand is degraded, while only one strand of the duplex is stably associated with RISC. The mature miRNA can interact with target mRNAs, containing partially complementary miRNA binding sites within the 3′UTR region, inducing translation repression, mRNA target cleavage, or mRNA deadenylation. The image was created using the image bank of Servier Medical Art (Available online: http://smart.servier.com/, accessed on 30 December 2022), licensed under a Creative Commons Attribution 3.0 Unported License (Available online: https://creativecommons.org/licenses/by/3.0/, accessed on 30 December 2022). RNA polymerase II or III: RNA Pol II/III; microRNA: miRNA; primary miRNA: pri-miRNA; ras-related nuclear protein: Ran; Guanosine-5′-triphosphate: GTP; precursor miRNA: pre-miRNA; RNA-induced silencing complex: RISC; messenger RNA: mRNA.

MiRNAs are small ncRNAs (~19–24 nucleotides), which typically lead to gene silencing by driving Argonaute (AGO) proteins to bind specific sites in the 3′UTR of mRNAs [85]. However, other target sites have been detected in the 5′UTR, in the coding sequence, and even in promoter regions. The miRNAs either block translation or degrade the target mRNA by process of “hetero-silencing”. The same miRNA can target different mRNAs; conversely, a single mRNA can be regulated by multiple miRNAs. The transcription of miRNAs takes place, especially by the work of RNA polymerase II, towards which they show a particular affinity thanks to the presence in the primary transcript of promoters that contain typical characteristics of RNA polymerase II [86]. The miRNAs originate from a long double-stranded transcript, the primary miRNA (pri-miRNA), which is recognized in the nucleus by the protein DGCR8 (Drosha’s partner). This protein is associated with Drosha, also called Ribonuclease III (RNase III), and directs its Drosha catalytic domain by cutting pri-miRNA and thus obtaining miRNA precursors (pre-miRNA) [87]. Pre-miRNAs are then carried to the cytoplasm by exportin-5, an export receptor which requires ras-related nuclear protein-Guanosine-5′-triphosphate (Ran-GTP) proteins for cargo binding. The nuclear export of pre-miRNAs is a crucial step in miRNA biogenesis. In particular, exportin-5 can recognize the double-stranded RNA of pre-miRNAs in a sequence-independent manner [88,89,90]. In the cytoplasm, pre-miRNA is further processed by RNase III endonuclease Dicer which removes the terminal loop, resulting in a mature RNA molecule of approximately 22 nucleotides called miRNA. Following cleavage, one RNA strand is degraded. The other strand is loaded, from the 5′ or 3′ end, into AGO protein to form ‘miRNA-induced silencing complex’ (miRISC), where mature miRNAs bind and regulate specific mRNAs. The name of mature miRNAs, 5p or 3p, depends on the directionality of the miRNA strand [91,92]. Post-transcriptional gene silencing can occur following different mechanisms depending on the complementarity between the miRNA and its mRNA target. One mechanism involves deadenylation by cap removal and exonucleolytic digestion of mRNA, a process that occurs when miRNAs bind perfectly complementary areas of their target mRNA. Instead, when miRNAs bind mRNA with imperfect complementarity, a translation block occurs, which can happen by repression of translation during the initial phase or during the elongation phase. Repression of translation by miRNAs can also occur, inducing premature ribosome detachment (Figure 1) [93].

However, in certain circumstances, miRNAs have also been reported to promote gene upregulation [94]. In addition, it has been revealed that miRNA abundance in the organism depends, at least in part, on their stability [95]. Indeed, the miRNAs secreted in extracellular fluids, such as blood, CSF, or saliva, may have potential as biomarkers for many diseases. MiRNA function is essential to promote development and biological processes, and their deregulation has been related to several pathological conditions [94]. In particular, these molecules are highly expressed in the CNS, which is involved in the development and homeostasis of the brain. Overall, miRNAs are essential for many biological functions within the neurons, including proliferation, apoptosis, and synaptic plasticity [96,97,98]. The aberrant expression of many miRNAs has been linked to impaired cognitive functions and memory loss in experimental models. The role of miRNAs in the neuropathogenesis of AD has been proved in several studies. However, further research is needed to also confirm in vitro and in vivo results in the human brain [99,100,101]. The dysfunction of miRNAs could affect AD progression by modulating neurodegeneration, neurotoxicity, and synaptic loss. Indeed, miRNA deregulation has been observed in the brain of AD patients compared to healthy controls, but their exact implication in AD pathogenesis is still unclear [102,103].

Of note, miRNA dysregulation could influence MAPK signaling during AD. Therefore, the use of miRNAs to regulate different genes involved in pathologies could be an interesting strategy to regulate neuronal homeostasis and allow neuronal circuits to respond adequately to environmental insults [104]. The miRNA’s ability to bind multiple mRNAs aroused interest as a potential therapeutic treatment. AD as a complex disorder could require a multi-targeted approach in order to inhibit different aspects of pathology. Growing data also suggest that miRNAs are critical regulators of pathophysiological processes [105,106]. However, the use of miRNAs could create potential problems due to their ability to modulate molecular pathways that could improve some pathological conditions but also have an influence on non-deregulated pathways [107]. Because they differ from conventional drugs, such as small molecule and protein drugs, which are also known to act primarily on protein targets, RNA-based therapies are considered to be the next generation of therapeutics [106,108]. First, RNA aptamers can produce pharmacological effects by blocking the activity of a particular protein target [109]. Second, to control a specific disease, antisense (asRNA), small interfering RNAs (siRNA), and miRNA can be created to specifically target functional mRNAs or ncRNAs [110]. Third, to treat a monogenic condition, guide RNAs (gRNAs) can be used to precisely alter the target sequences of a particular gene [111]. Currently, the mutual regulation between miRNAs and their target genes represents a challenge. Thus, in vivo studies on gene regulations mediated by miRNAs may have important implications for their clinical use [112]. Thus, RNA therapies have the potential to increase the number of therapeutic targets. At present, several pharmaceutical and biotech companies are working on possible therapeutics based on suppressing or re-establishing the concentration of specific miRNAs using, respectively, antagomiR (anti-miR) or miRNA mimics [113]. However, the use of miRNAs could increase significantly in subsequent years, which will contribute to the development of successful precision medicine and more personalized therapies.

## 4. The MAPK Pathway

**Figure 2:**
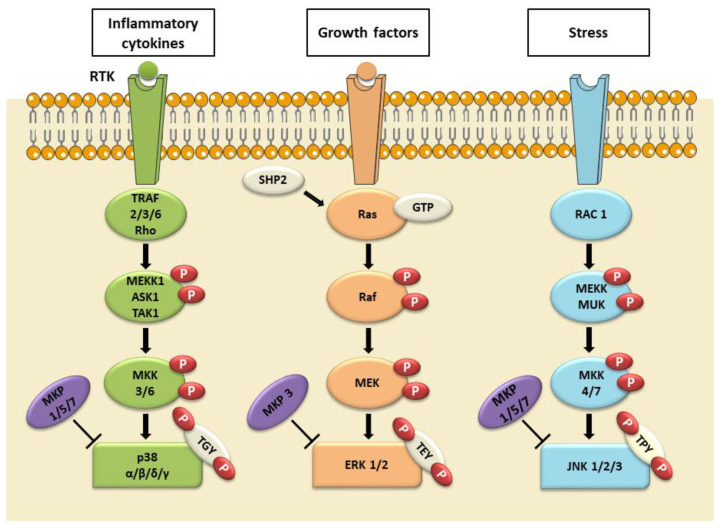
The MAPK signaling pathways. In mammalian cells, there are three well-known MAPK pathways: the ERK1/2, the c-JUN N-terminal kinase 1, 2, and 3 (JNK1/2/3), and the p38 α, β, δ, and γ MAPK pathways. ERK1/2 is activated in response to growth factors, hormones, and proinflammatory stimuli, while JNK1/2/3 and p38 α, β, δ, and γ are activated by cellular and environmental stresses, in addition to pro-inflammatory stimuli. The image was created using the image bank of Servier Medical Art (Available online: http://smart.servier.com/, accessed on 30 December 2022), licensed under a Creative Commons Attribution 3.0 Unported License (Available online: https://creativecommons.org/licenses/by/3.0/, accessed on 30 December 2022). Mitogen-activated protein kinases: MAPKs; c-Jun N-terminal kinase 1, 2, and 3: JNK1/2/3; extracellular signal-regulated kinases 1 and 2: ERK1/2; MAPK phosphatases 1, 3, 5, 7: MPK1/3/5/7; tumor necrosis factor receptor-associated factor: TRAF; SH2 containing protein tyrosine phosphatase-2: SHP2; Guanosine-5′-triphosphate: GTP; Rac Family Small GTPase 1: RAC1; MEK kinase 1: MEKK1; Apoptosis signal-regulating kinase 1: ASK1; transforming growth factor-β-activated kinase 1: TAK1; MAPK upstream kinase: MUK; MAP kinase kinase 3, 4, 6 and 7: MKK3/4/6/7; Thr-Gly-Tyr motif: TGY; Thr-Glu-Tyr motif: TEY; Thr–Pro–Tyr motif: TPY; receptor tyrosine kinase (RTK).

MAPKs are serine-threonine kinases that mediate cellular response to external stimuli through different transduction signals. MAPKs are ubiquitously expressed and evolutionarily conserved in eukaryotes [114,115]. The MAPK signaling pathway transduces signals through downstream phosphorylation of proteins from the membrane receptor to the cytoplasm and nucleus [116]. Activation of a MAPK cascade occurs in the form of consecutive phosphorylations, i.e., a Mitogen-Activated Protein Kinase Kinase Kinase (MAP3K) activates a Mitogen-Activated Protein Kinase (MEK), which then, in turn, activates a MAPK [114,115,117,118]. Phosphorylation events of MAPKs can be inactivated by MAPK phosphatases (MKPs) which dephosphorylate both phosphothreonine and phosphotyrosine residues present in MAPKs [117,119]. In mammals, three main groups of kinases have been characterized: ERK, JNK, and p38 MAPK. In general, ERK is activated by growth factors, while JNK and p38 are induced by cellular stress. Canonical activation of the ERK1 and ERK2 isoforms begins following the binding of a ligand to a receptor tyrosine kinase (RTK) present on the plasma membrane, followed by the activation of the small G protein, Ras. Next, Ras recruits and activates serine/threonine protein kinase Raf, a MAP3K, which activates MEK, which, in turn, phosphorylates both threonine and tyrosine residues within the TEY (Thr-Glu-Tyr) motif of MAPK and ERK1/2 [120,121]. Instead, p38 MAPK isoforms are activated by both stress and cytokines and play a key role in inflammatory responses [122,123]. In response to stress or cytokines, tumor necrosis factor receptor-associated factor (TRAF) 2/3/6 or Rho proteins activate a MAP3K, such as MEK kinase 1 (MEKK1), apoptosis signal-regulating kinase 1 (ASK1), or transforming growth factor-β-activated kinase 1 (TAK1). MAP3K, in turn, phosphorylates a MEK, MAP kinase kinase 3, or 6 (MKK3 or MKK6), which subsequently phosphorylates the TGY (Thr-Glu-Tyr) motif of the p38 MAPK isoforms (Figure 2) [124,125]. Thus, MAPKs are involved in many biological activities, including cell proliferation, differentiation, apoptosis, and survival [126,127,128,129,130]. The ERK/MAPK pathway sends developmental signals from upstream activators to downstream effectors, cytoplasmic and nuclear substrates, which also regulate several stages of neurodevelopment, such as neural induction, neural patterning, neurogenesis, and neurite outgrowth [131,132,133].

Therefore, due to the pleiotropic functions of the MAPK signaling cascade, its aberrant activations are known to be involved in numerous pathologies, including neurodegenerative diseases. Literature data suggest that ERK, JNK, and p38 MAPK are all implicated in AD, playing a role in various aspects of the disease, such as apoptosis, neuronal plasticity, neurotoxicity, and autophagy [134,135]. However, excessive ROS production occurs during early stage AD due to mitochondrial dysfunctions in neurons. It has been seen that MAPKs can be activated by oxidative stress in a number of different cell types [48,136]. Moreover, Aβ accumulation and Tau hyperphosphorylation, which affect neurons in AD, as well as neuroinflammation, have been associated with the MAPK cascade in several studies. ERK overactivation is known to increase Aβ production, while inhibition of the JNK pathway blocks c-Jun, caspase-2 (CASP-2), and caspase-3 (CASP-3) activation. In addition, p38 MAPK inhibition has shown neuroprotective effects against neuronal damage, suggesting its potential as a strategic treatment for AD [21,135,137,138].

## 5. Molecular Interactions of miRNAs and MAPKs in the Underlying Mechanisms of AD

### 5.1. Cross-Talk of miRNAs with MAPK Signaling Pathway in the Regulation of Tau and Aβ Protein Pathological Formation in AD

Several pieces of evidence have proven that miRNA deregulation in AD may promote Aβ and Tau pathology by modulating the MAPK pathway [139,140]. On the other hand, some studies have reported that the aberrant activation of MAPKs led to miRNA dysregulation with consequent neuronal damage [141,142]. Thus, the mechanisms through which miRNAs and MAPKs modulate each other, contributing to AD development, are still not entirely clear. In this regard, miR-148-3p reduced expression levels have been associated with the elevation of p38 MAPK by targeting Phosphatase and tensin homolog (PTEN) in the AD mice model [143]. Among MAPKs, p38 MAPK is known to be involved in Tau phosphorylation [144]. Thus, Zeng et al. [143] suggested that miR-148a-3p downregulation may increase Tau phosphorylation via the PTEN/p38 MAPK pathway in vivo. The authors showed that miR-148-3p levels were decreased in the serum of AD patients, but also in amyloid precursor protein/presenilin-1 (APP/PS1) and SAMP8 (senescence-accelerated mouse prone 8) transgenic mice brain tissue. The APP/PS1 and the SAMP8 mice were characterized by pathological AD typical features, β-amyloid production and cognitive decline, respectively [145]. However, the therapeutic potential of miR-148a-3p was also assessed by injection of miR-148a-3p mimics or PTEN siRNA in the cortex and hippocampus of APP/PS1 mice. Interestingly, Zeng et al. also found that miR-148a-3p overexpression improved AD cognitive deficit and decreased p-Tau. This neuroprotective effect was also confirmed in vitro using the APP^swe^ cell (SH-SY5Y cells transfected with the Swedish mutant form of human *APP*) model: the upregulation of miR-148a-3p reduced Aβ-induced injury by increasing cell viability and inhibiting Tau abnormal phosphorylation. Therefore, the data suggest the important role of miR-148a-3p in the progression of AD by indirect modulation of p38 MAPK signaling and Tau phosphorylation. The molecular mechanism induced by miR-148-3p could be used to ameliorate cognitive defects and neuronal degeneration [143].

Another significant deregulation is the overexpression of miR-342-3p, which has been identified in both post-mortem hippocampal samples from human AD patients and the murine AD model [146,147]. Fu et al. [148] proved that miR-342-3p upregulation exacerbated AD symptoms, as well as amyloid production and deposition in hippocampal tissues of triple transgenic AD (3xTg-AD) mice. This model is widely used to study AD since 3xTg-AD mice displayed both plaque and tangle pathology, as well as synaptic dysfunction, by expressing three dementia-related transgenes [149]. However, miR-342-3p inhibition with anti-miR improved cognitive deficit and decreased the Aβ-plaque burden in vivo, as revealed by immunohistochemical analysis. In accordance with the literature [140,150], Aβ stimulation increased JNK and ERK activation. It has been suggested that the miR-342-3p was acting as both target and modulator of Aβ-induced neuronal damage through JNK. However, miR-342-3p expression has been evaluated using different MAPK inhibitors after Aβ stimulation in HT22 cells. Only SP600125, a JNK inhibitor, could reverse miR-342-3p upregulation induced by Aβ exposure. Thus, Aβ might modulate miR-342-3p via the JNK pathway in vitro, but the increase in miR-342-3p levels could enhance JNK activation with a strong reduction of cellular vitality. In general, JNK is known to be involved in the regulation of apoptosis and survival signals in neurodegenerative diseases [151]. Therefore, data from this study confirm that hippocampal signal transduction derangement and neuronal apoptosis in AD result from the increased Aβ burden and chronic activation of the JNK cascade in a miR-342-3p-dependent manner. Consequently, intrahippocampal miR-342-3p inhibition could be a useful strategy to reduce Aβ plaques and improve learning and memory in AD patients [148].

According to another study, miR-125b, which is one of the most upregulated miRNAs in the brain of AD patients [152,153,154,155], interacted with MAPK signaling by inhibiting Dual Specificity Phosphatase 6 (DUSP6), also called MAPK phosphatase in vivo [156]. Thus, miR-125b overexpression led to enhanced phopho-p44/42-MAPK (p-ERK1/2) levels. Elevated p-ERK1/2 protein levels have been reported in the brain of both mice and humans with AD. Since ERK1/2 is known to phosphorylate Tau proteins on multiple sites through the Cyclin-dependent kinase 5 and its regulatory subunit p35 (cdk5/p35), the overactivation of these kinases may promote Tau pathology in AD. Based on the results, miR-125b overexpression exerted neurotoxic and pro-apoptotic effects, increasing memory and learning impairment, as shown in two behavioral assays of C57BL/6 wild-type (WT) mice. This suggests that the miR-125b/ERK axis may also lead to cognitive deterioration of cognitive functions in human patients with AD. The inhibition of miR-125b could be a new promising approach for AD management, but potential adverse side effects due to the reduction of miR-125b levels under baseline conditions should be investigated in future experiments [156].

The aberrant activation of ERK/MAPK was associated with Aβ pathology, as well as Tau phosphorylation, in other studies [150,157]. Growing evidence agrees that the miR-132/212 cluster is implicated in the neurophysiological process, including synaptic plasticity and memory formation [158,159,160]. Moreover, miR-132/212 was found to be downregulated in AD [161,162,163]. Hernandez-Rapp et al. [164] observed that the genetic deletion of the miR-132/212 cluster promoted amyloid aggregation and deposition in cortical and hippocampal tissues 3xTg-AD mice compared to the WT control, as well as the upregulation of ERK2 (MAPK1), Sirtuin 1 (Sirt1), and Tau proteins. In particular, MAPK1 was identified as a target of miR-132. These results were confirmed in vitro: the miR-132 overexpression caused the decrease of these genes’ expression in mouse Neuro2a cells expressing the Swedish mutant of *APP* and Δ9 mutant of *PSEN1* (Neuro2a APP^swe^/Δ9) and human HEK293 cells expressing the Swedish mutant of *APP* (HEK293-APP^swe^), with the consequent reduction of Aβ. Thus, the loss of miR-132/212 enhanced Tau phosphorylation, Aβ pathology, and cognitive impairment. Interestingly, miR-132 was found to be downregulated in human post-mortem tissues of AD cases compared to non-dement controls. Therefore, the miR-132/212 network could control various mechanisms of AD pathogenesis by also regulating Tau and Aβ pathology through ERK signaling. Indeed, ERK has been suggested to act upstream of Aβ generation by regulating BACE1 [164].

Another study [165] confirmed the neuroprotective effects of miR-132 by negatively regulating BACE1 and ERK activity in APP/PS1 mice. The use of a miR-132 mimic was proposed as a potential strategy to ameliorate AD progression. The strong link between miR-132, ERK1/2, Aβ, and Tau pathology has been assessed in the hippocampus of AD mice but also in the human AD cortex. Consistently, miR-132 downregulation has been verified. However, the authors did not find a direct correlation between miR-132 and Tau with respect to the article mentioned above. MiR-132 has been suggested to affect Tau phosphorylation in an indirect manner by inhibiting 1,4,5-triphosphate 3-kinase B (ITPKB) and ERK1/2 activity. It has been suggested that miR-132 downregulation was both a cause and a consequence of AD pathology. Therefore, the use of miR-132 mimics could be an interesting strategy to mitigate the ongoing neurodegenerative process in AD patients. Indeed, Aβ and Tau levels were found to decrease after intracerebral ventricular (ICV) injection with miR-132 mimic in AD mice [165].

On the other hand, Nagaraj et al. [166] identified miR-483-5p as a possible blood-based biomarker because it was found to be upregulated in the plasma of AD patients and also from the first symptoms, so-called prodromal AD patients. Using an in silico approach, miR-132-3p and miR-483-5p were compared. MiRNA molecular targets involved in the neuroprotective mechanisms were identified and subsequently confirmed in vitro using HEK293 and SK-N-MC cellular-based models. miR-483-5p upregulation may protect against AD pathology since it decreases Tau phosphorylation by reducing ERK1 and MAPK1 mRNA levels. CRISPR/Cas9-mediated genomic deletion in neonatal fibroblasts supported miR-483-5p binding to ERK1. This could represent a novel target for AD, but further experimental research is needed to better understand the miR-483-5p/ERK1/Tau interaction [166].

Moreover, ERK signaling modulation was also associated with another miRNA called miR-126 [167]. The overexpression of miR-126 increased Aβ_1-42_ toxicity in Tg6799 mice, a familial model of AD, through the downregulation of ERK and growth factor/Phosphatidyl Inositol 3-Kinase/Protein kinase B (PI3K/AKT) signaling. According to Kim et al. [168], even a small increase in miR-126 expression might affect growth factor activities in both normal neurons and neurons with disease-associated mutations. It must be considered that ERK signaling is involved not only in Tau phosphorylation but also in neuronal functionality and aging [169,170,171]. The inhibition of miR-126 has been associated with neuroprotective effects without compromising normal cell functions. Thus, miR-126 dysregulation has been suggested as a potential promoter of metabolic dysfunctions and toxicity during aging or neurodegenerative diseases by modulating PI3K and ERK signaling [168].

### 5.2. Molecular Interactions of miRNAs with the MAPK Signaling Pathway in the Oxidative Stress Modulation Underlying AD

It has been suggested that miRNAs deregulated by oxidative stress may contribute to AD development by regulating protein ubiquitination and phosphorylation through the MAPK signaling pathway [172,173,174,175].

According to Shunjiang Xu et al. [172], several miRNAs were upregulated in primary cultured hippocampal neurons after stimulation with H_2_O_2_, including miR-708, miR-296, miR-200c, miR-377, and miR-1190. The significantly increased expression of miR-708 was related to the process of cell apoptosis in gene ontology enrichment. Bioinformatics analysis revealed five target genes of miR-708 (*Map3k13*, *Kras*, *Rap1b*, *Nras*, and *Csf1*) that were predicted to affect MAPK signaling. Given its role in cell differentiation, synaptic plasticity, and learning, it was suggested that the deregulation of MAPK induced by miR-708 might contribute, at least in part, to synaptic loss during AD progression [172]. Using the same in vitro model, Zhang (2014) [173] showed other neuronal miRNAs that were modulated by oxidative stress in primary hippocampal neurons as well as the hippocampus of senescence-accelerated mice (SAM), SAMP8 and SAMP10 mice, respectively. These mice strains are widely used as models of AD, differing for some age-related pathological features such as memory and learning impairment or neurodegeneration. In this case, microarray results have proved that miR-329, miR-193b, miR-20a, miR-296, and miR-130b were upregulated after H_2_O_2_ stimulation. The authors suggested a correlation between miRNAs altered levels and the downregulation of neuronal genes in the AD brain. Indeed, enrichment analysis showed that miRNA upregulation could interfere with several biological processes, including cell growth or apoptosis. However, the Kyoto Encyclopedia of Genes and Genomes (KEGG) analysis of pathway enrichment revealed that miRNA alteration induced by oxidative stress could mainly impair the MAPK pathway, leading to synaptic loss and neuron death during AD. In this context, miR-20a is of particular interest: it could be involved in brain development by targeting markers such as Mitogen-activated protein kinase kinase kinase 12 (MAP3K12), influencing aging. In both studies, KEGG enrichment analysis provided that MAPK signaling was one of the main pathways to be impaired by oxidative stress-induced miRNAs. However, these results suggested that ROS production led to the dysregulation of both miRNAs and MAPK pathways, contributing to the pathology of AD [173].

Interestingly, miR-34c has been identified to be dysregulated in the hippocampus, plasma, and cerebrospinal fluid of patients with AD [176]. In this context, Shi et al. [175] investigated the expression patterns of miR-34c in oxidative–stressed hippocampal neurons and SAMP8 mice. The results showed that miR-34c was overexpressed in neurons treated with H_2_O_2_ or Aβ_1–42_, as well as in cortical and hippocampal regions of SAMP8 mice with aging. ROS production promoted JNK phosphorylation, which stimulated p53 protein accumulation and activation in vitro. It is well known that p53 activation leads to neuron loss during AD and that miR-34c is upregulated after p53 activation [177,178,179]. Consistently, miR-34c inhibition promoted cognitive decline and memory function by reducing Aβ-induced synaptic damage in SAMP8 mice. Thus, miR-34c was upregulated through the ROS-JNK-p53 pathway in the development of AD [175].

Other studies, suggested that several miRNAs can modulate oxidative stress by targeting different genes [180,181]. A study revealed that miR-132 could decrease oxidative stress and improve cognitive functions by targeting MAPK1 in AD rat models obtained after ICV injection with Aβ_25–35_ [174]. The overexpression of miR-132 was reported to reduce Nitric oxide synthase (iNOS) levels. Indeed, miR-132 expression was decreased in the hippocampus of rats with AD, while MAPK1 was upregulated as well as iNOS. It has been demonstrated that miR-132 overexpression or MAPK1 silencing decreased ROS and iNOS expression but upregulated superoxide dismutase (SOD) and glutathione peroxidase (GSH-Px) levels in the serum of AD rats. Thus, miR-132 could stimulate the antioxidant system and reduce the apoptosis rate by inactivating the MAPK pathway. The inhibition of MAPK1 interfered with p38 MAPK signaling, which has been associated with neuron apoptosis as a response to excessive ROS production. Indeed, p38 MAPK is known to play a role in cell death through the activation of several proteins, including p53, c-Jun and c-Fos, Bax, and CASP-3. The authors suggested that miR-132 may ameliorate cognitive functions, exerting neuroprotective effects by decreased p38 MAPK activity, oxidative stress, and, consequently, cognitive decline. These results may be used to better understand the molecular mechanisms of miR-132 in the pathogenesis of AD in order to develop novel clinical strategies [174].

### 5.3. MiRNAs via the MAPK Signaling Pathway Regulate Microglia-Mediated Neuroinflammation and Neuron Death

Different studies showed that miRNAs modulate the inflammatory response of activated microglia and neuronal apoptosis via targeting the MAPK signaling pathway [182,183].

Using both BV-2 and HT22 cells stimulated with Aβ as an in vitro AD model, Shang et al. [184] found that miR-590-5p mimic injection restored cell viability by improving proliferation. In addition, miR-590-5p levels were found to be reduced in the serum of both AD patients and APP/PS1 transgenic mice. miR-590-5p downregulation was suggested to contribute to the pathogenesis and progression of AD through the activation of the TNF Receptor Associated Factor 3 (TRAF3)/p38 MAPK pathway. According to the findings, the anti-apoptotic effect of miRNA might partly be due to the inhibition of Pellino-1 (PELI1), which increased TRAF3 but decreased the expression as well as the phosphorylation of p38 MAPK and ERK1/2. Consistently, activation of the p38 MAPK pathway has been previously found in the brain tissue of AD cases [184].

Several findings proved the importance of cell cycle suppression for neuronal survival [185,186,187]. Although mature neurons inhibit the cell cycle in physiological conditions, it could be induced again by neurotoxic agents such as Aβ_42_ [188]. Aβ_42_ can promote cell cycle re-entry by promoting aberrant MEK-ERK signaling in neurons [189]. This excessive activation leads not to cell mitosis but to DNA replication and apoptosis. Modi et al. [190] evidenced that the MEK-ERK hyperactivated pathway led to overexpression of CyclinD1 by reducing miR-34a levels in a Tap73-dependent manner. In silico analysis revealed that miR-34a targeted the 3′UTR region of the CyclinD1 gene, showing neuroprotective effects by suppressing cell cycle re-entry and apoptosis in vitro. Interestingly, miR-34a expression was altered in Aβ_42_-induced cortical neurons from rats or APP/PS1 mice. Furthermore, its expression was previously found to be higher during neuronal differentiation, supported by p53 family member Tap73. However, cell cycle re-entry has been mainly studied in Alzheimer’s since it may contribute to neuron loss in patients [191]. Based on the finding data, cell cycle-related neuronal apoptosis (CRNA) may be regulated by miR-34a and ERK signaling in neurons [190].

In another study, miR-326 was found to reduce apoptosis by inactivating the JNK signaling pathway via Vav Guanine Nucleotide Exchange Factor 1 (VAV1) in the APP^swe^/PS1 double transgenic mice model of AD. The authors provided that miR-326 ameliorated AD progression and enhanced cell viability since miR-326 overexpression or/and JNK inhibition decreased Aβ_1–40_ and Aβ_1–42_ contents in brain tissues of AD mice. These results suggested that miR-326 could reduce Aβ deposition and Tau phosphorylation by inhibiting the JNK signaling pathway. Indeed, miR-326 mimic lentiviral vector or/and SP600126 injections improved cognitive deficit in AD mice compared to WT control, as revealed by the Morris water maze test. All findings have been predicted by bioinformatics analysis and subsequently confirmed experimentally [192].

According to different studies, miR-155 exerts pro-inflammatory activity by modulating certain components of the innate immune system [193,194]. MiR-155 has been associated with neuroinflammation, which occurs in the early stages of AD. Indeed, Guedes et al. showed that the c-Jun transcription factor (c-Jun) could regulate neuroinflammation by increasing miR-155 expression before extracellular Aβ deposition in vivo. Aβ production, has been associated with JNK activation and, as a consequence, with the downstream effector c-Jun. However, miR-155 upregulation induced by intracellular Aβ peptides was associated with glial cell activation and higher levels of cytokines in the hippocampal and cortical regions of 12-month-old 3xTg AD mice. The results were also confirmed in vitro by measuring miR-155 levels in Aβ-treated N9 microglia and astrocyte primary cultures. Thus, the authors proposed c-Jun silencing as a potential strategy to control AD pathogenesis and progression. Furthermore, modulation of miRNA expression in the brain by targeting glial cells with the aim of decreasing miR-155 levels could be an interesting strategy being explored in the context of AD [195].

Microglia have a central role in the maintenance of the CNS, but Aβ deposition can stimulate apoptosis in these cells [196]. Wan et al. demonstrated that miR-191-5p transfection in microglial cells reduced ERK1/2 and p38 MAPK activity by targeting the upstream MAP3K12 effector, which has been related to neuron stress response apoptosis and AD neurodegeneration. In particular, miR-191-5p overexpression was associated with BACE1 and Tau-5 (AD’s markers) downregulation, with a consequent decrease in the apoptosis rate in microglial cells. Indeed, miR-191-5p was found to be downregulated in hippocampal sections from APP/PS1 mice, suggesting that the deregulation of this miRNA may play a role in AD progression. However, miR-191-5p seems to alleviate microglial cell injury by targeting the MAP3K12/MAPK signaling pathway, but further experiments should be performed in vivo in order to validate the results seen in vitro [197].

**Figure 3:**
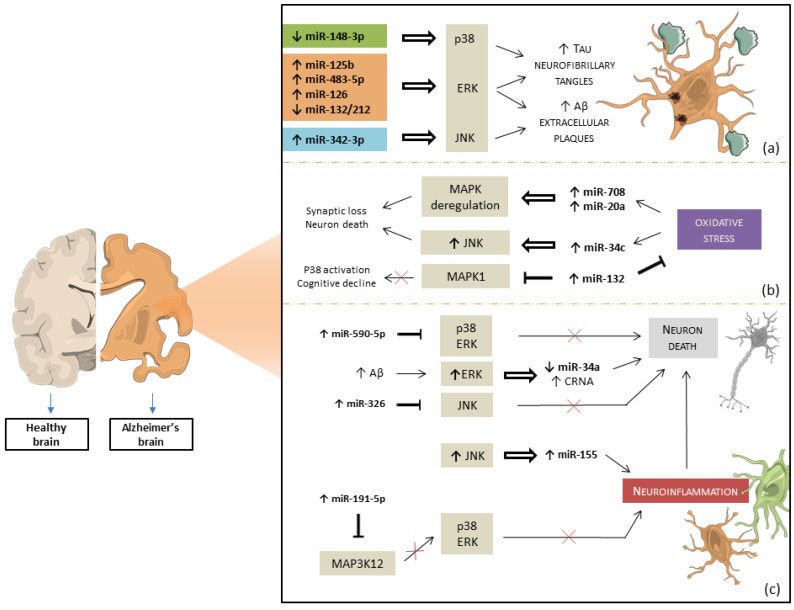
miRNAs and MAPKs interaction in Alzheimer’s disease. (**a**) The overexpression or the downregulation of miRNAs can act on p38 MAPK, JNK, and ERK in a direct or indirect way, exacerbating NFT and amyloid extracellular plaques in AD neurons. (**b**) Oxidative stress enhances miRNAs expression during AD, causing deregulation in the MAPK pathway and, consequently, synaptic and neuron loss. The overexpression of miR-132 decreases oxidative stress, p38 MAPK, and cognitive decline by targeting MAPK1. (**c**) The Aβ depositions induce ERK activation, which leads to a decrease in miR-34a expression levels and, thus, to neuronal death due to the CRNA. The overexpression of miR-590-5p and miR-326 plays an anti-apoptotic effect through indirect inhibition of p38 MAPK, ERK, and JNK activation. JNK mediates downstream miR-155 upregulation and promotes neuroinflammation during AD. The overexpression of miR-191-5p reduces microglia injury through the decrease in p38 MAPK and ERK activity by targeting the upstream MAP3K13 effector. Neuroinflammation plays an important role in the onset and progression of neurodegeneration and neuronal loss in neurodegenerative diseases. The image was created using the image bank of Servier Medical Art (Available online: http://smart.servier.com/, accessed on 30 December 2022), licensed under a Creative Commons Attribution 3.0 Unported License (Available online: https://creativecommons.org/licenses/by/3.0/, accessed on 30 December 2022). microRNAs: miRNAs; Mitogen-activated protein kinases: MAPKs; c-JUN N-terminal kinase: JNK; extracellular signal-regulated kinases: ERK; Neurofibrillary tangles: NFT; Alzheimer’s disease: AD; Mitogen-Activated Protein Kinase 1: MAPK1; reactive oxygen species: ROS; amyloid-β: Aβ; Transcription factor Jun: c-Jun; Mitogen-Activated Protein Kinase Kinase Kinase 12: MAP3K12; cell cycle-related neuronal apoptosis (CRNA).

The results are summarized in Table 1, which includes in vitro and in vivo evidence of miRNAs and MAPKs interaction in AD, as shown in Figure 3. Experimental studies have proven that miRNAs or MAPK signaling deregulation can contribute to AD progression by modulating Aβ and Tau pathology, oxidative stress, neuroinflammation, and neuron death. Moreover, the modulation of the MAPK pathway using miRNAs mimic injection or silencing may improve cognitive decline and neurodegeneration, as revealed in AD animal models. Thus, miRNAs are involved in MAPK signaling modulation, and the molecular interactions between miRNAs and the MAPKs pathway seem to have a potential for both diagnostics and therapeutics of AD.

## 6. Conclusions

The molecular interactions between miRNAs and MAPKs during AD may provide new research insights for understanding AD pathology. This review summarizes a number of recent findings which provide promising results on the therapeutic side. Based on obtained data, it was found that miR-125b upregulation led to memory and learning impairment by increasing p-ERK levels. At the same time, miR-132 showed neuroprotective effects by influencing ERK/MAPK1 activity, with the consequent reduction of both Aβ and Tau pathology hallmarks, as well as oxidative stress in AD animal models. Additionally, several pieces of evidence suggest that interaction between miRNAs and the JNK pathway may contribute to neuron death in AD. Whereas miRNAs have a multi-targeting ability, the modulation of MAPK signaling by acting on miRNA expression seems to improve cognitive decline in AD animal models. Using bioinformatics technologies and in vivo strategies could facilitate the development of novel approaches in both diagnostics and therapeutic fields for AD. However, further investigations should be conducted to better investigate these molecular interactions in order to replicate and, possibly, translate them into clinical applications.

## Figures and Tables

**Table 1:** Summary of included AD experimental models that highlight the role of miRNAs and MAPKs in the pathological mechanisms of disease.

Micro-RNA	Models	MAPK Interaction	Results	Ref.
Cross-talk of miRNAs with MAPKs signaling pathway in the regulation of Tau and Aβ Protein pathological formation in AD				
miR-148-3p	APP/PS1 and SAMP8 mice	p38 MAPK	MiR-148-3p downregulation led to PTEN inhibition, and p38 MAPK increased levels; miR-148-3p overexpression improved AD cognitive deficit and decreased Tau phosphorylation in vivo; neuroprotective effects against Aβ-induced injury were also observed in vitro.	[143]
	APP^swe^ cells			
miR-342-3p	3xTg-AD mice	JNK	MiR-342-3p upregulation exacerbated AD symptoms in vivo, while miR-342-3p inhibition improved cognitive deficit and decreased Aβ-plaque burden in vivo. SP600125 reversed miR-342-3p upregulation in vitro; instead, miR-342-3p increased JNK activation.	[148]
	HT22 cells			
miR-125b	Human brain samples; C57BL/6 mice	ERK signaling	MiR-125b was upregulated in AD brain samples, while the miR-125b mimic injection impaired learning and memory in vivo. The miR-125b upregulation increased Tau phosphorylation, CASP-3/7, ERK1/2, and cdk5/p35 levels in vitro. Conversely, miR-125b knockdown increased cell viability and reduced Tau levels in vitro.	[156]
	Rat primary hippocampal neurons			
miR-132	Human brain samples; MiR-132 knockout 3xTg-AD mice	ERK signaling	Genetic deletion of miR-132 increased Sirt1, ERK2, and Tau levels and promoted Aβ pathology; indeed, miR-132 was observed to be downregulated in human AD brains. Conversely, miR-132 upregulation decreased Sirt1 and Aβ_40/42_ levels in vitro.	[164]
	Neuro2a-APP^swe^; HEK293-APP^swe^ cells			
miR-132	Human brain samples; Tg (Thy1-APP^swe^, Thy1-PSEN1*L166P) mice	ERK signaling	AntagomiR-132 increased while miR-132 mimic injection de-creased Aβ levels and Tau phosphorylation in mouse hippocampus. The miR-132 downregulation increased ITPKB, BACE1, and ERK1/2 levels in mice. Interestingly, miR-132 was reduced in the AD human brain, but ERK1/2, p-Tau, and ITPKB were elevated.	[165]
miR-483-5p	HEK293 and SK-N-MC cells; neonatal human dermal fibroblasts for CRISPR/Cas9 genomic deletion of miR-483-5p	ERK signaling	miR-483-5p targeted ERK1 mRNA, decreasing p-ERK1/2 and, consequently, p-TAU levels; ERK1 expression increased after miR-483-5p deletion.	[166]
miR-126	Cortical and hippocampal primary cultures from rat embryos	ERK signaling	miR-126 increased Aβ_1–42_ toxicity, promoted neurite sprouting, and modulated the neuroprotective effect of IGF-1, NGF, BDNF, and sAPPα; ERK and PI3K/AKT signaling were downregulated in miR-126 overexpressing neurons; the inhibition of miR-126 had neuroprotective effects.	[168]
	Tg6799 mice or littermate control			
Molecular interactions of miRNAs with the MAPKs signaling pathway in the oxidative stress modulation underlying AD				
miR-708	Primary hippocampal neuron cultures from SAMR1 mice embryos	*Map3k13*, *Kras*, *Rap1b*, *Nras* and *Csf1*	miR-708 and miR-135b were significant upregulated in the oxidative stress in vitro model; miR-708 targeted five genes of the MAPK signaling pathway according to the KEGG pathway.	[172]
miR-20a	Primary hippocampal neuron cultures from SAMR1 mice embryos; hippocampus tissues of SAMR1, SAMP8 and SAMP10 mice	MAPK signaling	miR-329, miR-193b, miR-20a miR-296, and miR-130b were all upregulated in H_2_O_2_-induced cells; all co-regulated miRNAs affected MAPK signaling pathway according to the KEGG pathway; miR-20a may be involved in aging according to literature, by targeting APP and reducing MAP3K12.	[173]
MiR-34c	SAMP8 and SAMR1 mice; primary hippocampal neurons	JNK	miR-34c levels were increased in the blood of aMCI patients, in AD mice, and cells stimulated with H_2_O_2_ or Aβ_42_; miR-34c upregulation was age-related and depended on the ROS-JNK-p53 upstream pathway; miR-34c targeted SYT1; antagomiR-34c administration improved memory function in SAMP8 mice.	[175]
	HT22 cells; human embryonic kidney 293A cells			
miR-132	ICV injection of Aβ_25/35_ in rats	MAPK1	MAPK1, p-MAPK1 and iNOS levels increased in AD brain but decreased after miR-132 overexpression or siMAPK1; cognitive defects and pathological changes were lower with miR-132 mimic or siMAPK1; SOD and GSH-Px increased while AChE, ROS, and MDA decreased with miR-132 mimic or siMAPK1; miR-132 decreased apoptosis rate.	[174]
	SHSY5Y cells treated with H_2_O_2_; HEK-293 transfected with lentivirus vector			
MiRNAs via the MAPK signaling pathway regulate microglia-mediated neuroinflammation and neuron death				
miR-590-5p	B6C3-Tg (APP^swe^, PSEN1dE9)/Nju mice	ERK signaling, p38 MAPK	miR-590-5p is downregulated in the serum of AD patients and brain tissues or serum from mice; miR-590-5p overexpression partly reduced ERK1/2 and p38 MAPK expression and increased Traf3 in Aβ-induced cells.	[184]
	BV2 microglial cells; HT22 cells			
miR-34a	B6C3-Tg (APP^swe^, PSEN1dE9) 85Dbo/J; cortical neuronal culture from Sprague Dawley rats or APP/PS1 mice embryos	ERK signaling	MiR-34a expression increased during differentiation in vitro by targeting cyclin D1; Aβ decreased miR-34a levels in cortical neurons and enhanced Cyclin D1; miR-34a was neuroprotective by prevented CRNA; miR-34a downregulation in AD models was dependent on the MEK-ERK pathway.	[190]
	SHSY5Y cells			
mir-326	APP^swe^/PS1d E9 double Tg mice	JNK	miR-326 overexpression improved cognitive functions in mice; mir-326 downregulation led to AD progression by activating JNK signaling through VAV1; miR-326 overexpression reduced Aβ accumulation and neuron apoptosis by targeting VAV1 and inhibiting JNK signaling in AD mice; miR-326 induced cell cycle and increased cell viability in AD mice.	[192]
miR-155	3xTg AD mice	JNK, c-Jun	Astrocytes and microglia populations were increased in AD mice; glial pro-inflammatory phenotype but no Aβ extracellular deposition were observed in the brain of 12-month-old AD mice; IL-6, IFN-β, and miR-155 levels were upregulated in AD mice compared to WT; LPS led to increasing miR-155 levels in vitro; IL-6 and IFN-β upregulation depended on Aβ in vitro; c-Jun contributed to miR-155 upregulation following glial exposure to Aβ.	[195]
	N9 microglia cells; Astrocyte primary cultures			
miR-191-5p	APP/PS1 mice	MAP3K12, ERK signaling, p38 MAPK	MiR-191-5p was downregulated in the hippocampus of AD mice; miR-191-5p overexpression relieved Aβ_1–42_-induced microglia injury by targeting MAP3K12 and inactivating MAPK signaling.	[197]
	Primary microglia			

Mitogen-activated protein kinase: MAPK; MicroRNAs: miRNAs; Phosphatase and tensin homolog: PTEN; Alzheimer’s disease: AD; Amyloid precursor protein/presenilin-1: APP/PS1; Senescence accelerated mouse prone: SAMP; SH-SY5Y cells transfected with the Swedish mutant form of human APP: APPswe cell; Amyloid-β: Aβ; c-Jun amino-terminal kinase: JNK; Triple transgenic AD: 3xTg-AD; Transgenic: Tg; Sirtuin 1: Sirt1; Neuro2a cells expressing the Swedish mutant of APP and Δ9 mutant of PSEN1: Neuro2a APPswe/Δ9; HEK293 cells expressing the Swedish mutant of APP: HEK293-APPswe; 1,4,5-trisphosphate 3-kinase B: ITPKB; β-secretase 1: BACE1; Intracerebroventricular: ICV; Caspase: CASP; PhosphatidylInositol 3-Kinase: PI3K; Protein kinase B: AKT; insulin-like growth factor-1: IGF-1; nerve growth factor: NGF; brain-derived neurotrophic factor: BDNF; Senescence accelerated mouse-resistant/1: SAMR1; Mitogen-activated protein kinase kinase kinase 13: MAP3K13; Kirsten rat sarcoma virus: Kras; Ras-related protein Rap-1b: Rap1b; Neuroblastoma RAS viral oncogene homolog: NRAS; Colony Stimulating Factor 1: Csf1; Kyoto Encyclopedia of Genes and Genomes: KEGG; Hydrogen peroxide: H2O2; co-injections of APPswe and PS1ΔE9 plasmids on a C57BL/6 J genetic background: APPswe/PS1ΔE9 transgenic mice; Mitogen-activated protein kinase 1: MAPK1; Amnestic mild cognitive impairment: aMCI; Reactive oxygen species: ROS; Synaptotagmin 1: SYT1; double transgenic mice expressing a chimeric mouse/human amyloid precursor protein (Mo/HuAPP695swe) and a mutant human presenilin 1 (PS1-dE9): B6C3-Tg (APPswe, PSEN1dE9); Extracellular signal-regulated kinase: ERK; TNF Receptor Associated Factor 3: TRAF3; Cell cycle-related neuronal apoptosis: CRNA; Mitogen-activated protein kinase kinase: MEK; Vav Guanine Nucleotide Exchange Factor 1: VAV1; Interleukin 6: IL-6; Interferon-β: IFN-β.

## Data Availability

Not applicable.

## References

[1] Lynch C. (2020). World alzheimer report 2019: Attitudes to dementia, a global survey: Public health: Engaging people in adrd research. Alzheimer’s Dement..

[2] Grøntvedt G.R., Schröder T.N., Sando S.B., White L., Bråthen G., Doeller C.F. (2018). Alzheimer’s disease. Curr. Biol..

[3] Bejanin A., Schonhaut D.R., La Joie R., Kramer J.H., Baker S.L., Sosa N., Ayakta N., Cantwell A., Janabi M., Lauriola M. (2017). Tau pathology and neurodegeneration contribute to cognitive impairment in alzheimer’s disease. Brain.

[4] DeTure M.A., Dickson D.W. (2019). The neuropathological diagnosis of alzheimer’s disease. Mol. Neurodegener..

[5] Prendecki M., Florczak-Wyspianska J., Kowalska M., Ilkowski J., Grzelak T., Bialas K., Kozubski W., Dorszewska J. (2019). Apoe genetic variants and apoe, mir-107 and mir-650 levels in alzheimer’s disease. Folia Neuropathol..

[6] Liu Y., Cheng X., Li H., Hui S., Zhang Z., Xiao Y., Peng W. (2022). Non-coding rnas as novel regulators of neuroinflammation in alzheimer’s disease. Front. Immunol..

[7] Qin H., Hu C., Zhao X., Tian M., Zhu B. (2023). Usefulness of candidate mrnas and mirnas as biomarkers for mild cognitive impairment and alzheimer’s disease. Int. J. Neurosci..

[8] Bhaskaran M., Mohan M. (2014). Micrornas: History, biogenesis, and their evolving role in animal development and disease. Vet. Pathol..

[9] Zhao Y., Pogue A.I., Lukiw W.J. (2015). Microrna (mirna) signaling in the human cns in sporadic alzheimer’s disease (ad)-novel and unique pathological features. Int. J. Mol. Sci..

[10] Wang J., Cao Y., Lu X., Wang T., Li S., Kong X., Bo C., Li J., Wang X., Ma H. (2020). Micrornas and nervous system diseases: Network insights and computational challenges. Brief. Bioinform..

[11] Vergallo A., Lista S., Zhao Y., Lemercier P., Teipel S.J., Potier M.-C., Habert M.-O., Dubois B., Lukiw W.J., Hampel H. (2021). Mirna-15b and mirna-125b are associated with regional aβ-pet and fdg-pet uptake in cognitively normal individuals with subjective memory complaints. Transl. Psychiatry.

[12] Bazrgar M., Khodabakhsh P., Prudencio M., Mohagheghi F., Ahmadiani A. (2021). The role of microrna-34 family in alzheimer’s disease: A potential molecular link between neurodegeneration and metabolic disorders. Pharmacol. Res..

[13] Li Q.S., Cai D. (2021). Integrated mirna-seq and mrna-seq study to identify mirnas associated with alzheimer’s disease using post-mortem brain tissue samples. Front. Neurosci..

[14] Wang M., Qin L., Tang B. (2019). Micrornas in alzheimer’s disease. Front. Genet..

[15] Yoon S., Kim S.E., Ko Y., Jeong G.H., Lee K.H., Lee J., Solmi M., Jacob L., Smith L., Stickley A. (2022). Differential expression of micrornas in alzheimer’s disease: A systematic review and meta-analysis. Mol. Psychiatry.

[16] Konovalova J., Gerasymchuk D., Parkkinen I., Chmielarz P., Domanskyi A. (2019). Interplay between micrornas and oxidative stress in neurodegenerative diseases. Int. J. Mol. Sci..

[17] Idda M.L., Munk R., Abdelmohsen K., Gorospe M. (2018). Noncoding rnas in alzheimer’s disease. Wiley Interdiscip. Rev. RNA.

[18] Kim C., Kang D., Lee E.K., Lee J.-S. (2017). Long noncoding rnas and rna-binding proteins in oxidative stress, cellular senescence, and age-related diseases. Oxid. Med. Cell. Longev..

[19] Johnson E.C., Carter E.K., Dammer E.B., Duong D.M., Gerasimov E.S., Liu Y., Liu J., Betarbet R., Ping L., Yin L. (2022). Large-scale deep multi-layer analysis of alzheimer’s disease brain reveals strong proteomic disease-related changes not observed at the rna level. Nat. Neurosci..

[20] Perluigi M., Barone E. (2022). Aberrant protein networks in alzheimer disease. Nat. Rev. Neurol..

[21] Du Y., Du Y., Zhang Y., Huang Z., Fu M., Li J., Pang Y., Lei P., Wang Y.T., Song W. (2019). Mkp-1 reduces aβ generation and alleviates cognitive impairments in alzheimer’s disease models. Signal Transduct. Target. Ther..

[22] Chakraborty C., Sharma A.R., Patra B.C., Bhattacharya M., Sharma G., Lee S.S. (2016). Micrornas mediated regulation of mapk signaling pathways in chronic myeloid leukemia. Oncotarget.

[23] Ramírez-Moya J., Santisteban P. (2019). Mirna-directed regulation of the main signaling pathways in thyroid cancer. Front. Endocrinol..

[24] Masliah-Planchon J., Garinet S., Pasmant E. (2016). Ras-mapk pathway epigenetic activation in cancer: Mirnas in action. Oncotarget.

[25] Slattery M.L., Mullany L.E., Sakoda L.C., Wolff R.K., Samowitz W.S., Herrick J.S. (2018). The mapk-signaling pathway in colorectal cancer: Dysregulated genes and their association with micrornas. Cancer Inform..

[26] Falcicchia C., Tozzi F., Arancio O., Watterson D.M., Origlia N. (2020). Involvement of p38 mapk in synaptic function and dysfunction. Int. J. Mol. Sci..

[27] Origlia N., Arancio O., Domenici L., Yan S.S. (2009). Mapk, beta-amyloid and synaptic dysfunction: The role of rage. Expert Rev. Neurother..

[28] Correa S.A., Eales K.L. (2012). The role of p38 mapk and its substrates in neuronal plasticity and neurodegenerative disease. J. Signal Transduct..

[29] Cargnello M., Roux P.P. (2011). Activation and function of the mapks and their substrates, the mapk-activated protein kinases. Microbiol. Mol. Biol. Rev..

[30] Pei J.J., Braak E., Braak H., Grundke-Iqbal I., Iqbal K., Winblad B., Cowburn R.F. (2001). Localization of active forms of c-jun kinase (jnk) and p38 kinase in alzheimer’s disease brains at different stages of neurofibrillary degeneration. J. Alzheimer’s Dis. JAD.

[31] Troy C.M., Rabacchi S.A., Xu Z., Maroney A.C., Connors T.J., Shelanski M.L., Greene L.A. (2001). Beta-amyloid-induced neuronal apoptosis requires c-jun n-terminal kinase activation. J. Neurochem..

[32] Zhu X., Mei M., Lee H.G., Wang Y., Han J., Perry G., Smith M.A. (2005). P38 activation mediates amyloid-beta cytotoxicity. Neurochem. Res..

[33] Alzheimer’s Association (2022). 2022 alzheimer’s disease facts and figures. Alzheimer’s Dement. J. Alzheimer’s Assoc..

[34] Hampel H., Hardy J., Blennow K., Chen C., Perry G., Kim S.H., Villemagne V.L., Aisen P., Vendruscolo M., Iwatsubo T. (2021). The amyloid-β pathway in alzheimer’s disease. Mol. Psychiatry.

[35] Hardy J.A., Higgins G.A. (1992). Alzheimer’s disease: The amyloid cascade hypothesis. Science.

[36] Müller U.C., Deller T., Korte M. (2017). Not just amyloid: Physiological functions of the amyloid precursor protein family. Nat. Rev. Neurosci..

[37] Gupta A., Goyal R. (2016). Amyloid beta plaque: A culprit for neurodegeneration. Acta Neurol. Belg..

[38] Zhang Y.-W., Thompson R., Zhang H., Xu H. (2011). App processing in alzheimer’s disease. Mol. Brain.

[39] Doran E., Keator D., Head E., Phelan M.J., Kim R., Totoiu M., Barrio J.R., Small G.W., Potkin S.G., Lott I.T. (2017). Down syndrome, partial trisomy 21, and absence of alzheimer’s disease: The role of app. J. Alzheimer’s Dis..

[40] Parsons R.B., Austen B. (2007). Protein–protein interactions in the assembly and subcellular trafficking of the bace (β-site amyloid precursor protein-cleaving enzyme) complex of alzheimer’s disease. Biochem. Soc. Trans..

[41] Querfurth H.W., LaFerla F.M. (2010). Mechanisms of disease. N. Engl. J. Med..

[42] Zhang H., Ma Q., Zhang Y.w., Xu H. (2012). Proteolytic processing of alzheimer’s β-amyloid precursor protein. J. Neurochem. Rev..

[43] Näslund J., Haroutunian V., Mohs R., Davis K.L., Davies P., Greengard P., Buxbaum J.D. (2000). Correlation between elevated levels of amyloid β-peptide in the brain and cognitive decline. JAMA.

[44] Knopman D.S., Amieva H., Petersen R.C., Chételat G., Holtzman D.M., Hyman B.T., Nixon R.A., Jones D.T. (2021). Alzheimer disease. Nat. Rev. Dis. Prim..

[45] Lindwall G., Cole R.D. (1984). Phosphorylation affects the ability of tau protein to promote microtubule assembly. J. Biol. Chem..

[46] Iqbal K., Alonso A.d.C., Chen S., Chohan M.O., El-Akkad E., Gong C.-X., Khatoon S., Li B., Liu F., Rahman A. (2005). Tau pathology in alzheimer disease and other tauopathies. Biochim. Biophys. Acta (BBA)-Mol. Basis Dis..

[47] Zhang H., Wei W., Zhao M., Ma L., Jiang X., Pei H., Cao Y., Li H. (2021). Interaction between aβ and tau in the pathogenesis of alzheimer’s disease. Int. J. Biol. Sci..

[48] Misrani A., Tabassum S., Yang L. (2021). Mitochondrial dysfunction and oxidative stress in alzheimer’s disease. Front. Aging Neurosci..

[49] Nunomura A., Castellani R.J., Zhu X., Moreira P.I., Perry G., Smith M.A. (2006). Involvement of oxidative stress in alzheimer disease. J. Neuropathol. Exp. Neurol..

[50] Leng F., Edison P. (2021). Neuroinflammation and microglial activation in alzheimer disease: Where do we go from here?. Nat. Rev. Neurol..

[51] Kempuraj D., Thangavel R., Natteru P.A., Selvakumar G.P., Saeed D., Zahoor H., Zaheer S., Iyer S.S., Zaheer A. (2016). Neuroinflammation induces neurodegeneration. J. Neurol. Neurosurg. Spine.

[52] Kinney J.W., Bemiller S.M., Murtishaw A.S., Leisgang A.M., Salazar A.M., Lamb B.T. (2018). Inflammation as a central mechanism in alzheimer’s disease. Alzheimer’s Dement..

[53] Breijyeh Z., Karaman R. (2020). Comprehensive review on alzheimer’s disease: Causes and treatment. Molecules.

[54] Arvanitakis Z., Shah R.C., Bennett D.A. (2019). Diagnosis and management of dementia: Review. JAMA.

[55] Hung S.-Y., Fu W.-M. (2017). Drug candidates in clinical trials for alzheimer’s disease. J. Biomed. Sci..

[56] Scheltens P., De Strooper B., Kivipelto M., Holstege H., Chételat G., Teunissen C.E., Cummings J., van der Flier W.M. (2021). Alzheimer’s disease. Lancet.

[57] Crews L., Masliah E. (2010). Molecular mechanisms of neurodegeneration in alzheimer’s disease. Hum. Mol. Genet..

[58] Piaceri I., Nacmias B., Sorbi S. (2013). Genetics of familial and sporadic alzheimer’s disease. Front. Biosci.-Elite.

[59] Campion D., Dumanchin C., Hannequin D., Dubois B., Belliard S., Puel M., Thomas-Anterion C., Michon A., Martin C., Charbonnier F. (1999). Early-onset autosomal dominant alzheimer disease: Prevalence, genetic heterogeneity, and mutation spectrum. Am. J. Hum. Genet..

[60] Kim J., Basak J.M., Holtzman D.M. (2009). The role of apolipoprotein e in alzheimer’s disease. Neuron.

[61] Pellegrini C., Fornai M., D’Antongiovanni V., Antonioli L., Bernardini N., Derkinderen P. (2022). The intestinal barrier in disorders of the central nervous system. Lancet Gastroenterol. Hepatol..

[62] Askarova S., Umbayev B., Masoud A.-R., Kaiyrlykyzy A., Safarova Y., Tsoy A., Olzhayev F., Kushugulova A. (2020). The links between the gut microbiome, aging, modern lifestyle and alzheimer’s disease. Front. Cell. Infect. Microbiol..

[63] Zhu S., Jiang Y., Xu K., Cui M., Ye W., Zhao G., Jin L., Chen X. (2020). The progress of gut microbiome research related to brain disorders. J. Neuroinflamm..

[64] Krishaa L., Ng T.K.S., Wee H.N., Ching J. (2023). Gut-brain axis through the lens of gut microbiota and their relationships with alzheimer’s disease pathology: Review and recommendations. Mech. Ageing Dev..

[65] Collins S.M., Surette M., Bercik P. (2012). The interplay between the intestinal microbiota and the brain. Nat. Rev. Microbiol..

[66] Pearce S.C., Coia H.G., Karl J., Pantoja-Feliciano I.G., Zachos N.C., Racicot K. (2018). Intestinal in vitro and ex vivo models to study host-microbiome interactions and acute stressors. Front. Physiol..

[67] Dorszewska J., Hurła M., Banaszek N., Kobylarek D., Piekut T., Kozubski W. (2022). From infection to inoculation: Expanding the microbial hypothesis of alzheimer’s disease. Curr. Alzheimer Res..

[68] Nagarajan A., Srivastava H., Morrow C.D., Sun L.Y. (2023). Characterizing the gut microbiome changes with aging in a novel alzheimer’s disease rat model. Aging.

[69] van Olst L., Roks S.J., Kamermans A., Verhaar B.J., van der Geest A.M., Muller M., van der Flier W.M., de Vries H.E. (2021). Contribution of gut microbiota to immunological changes in alzheimer’s disease. Front. Immunol..

[70] Dinan T.G., Cryan J.F. (2017). Gut instincts: Microbiota as a key regulator of brain development, ageing and neurodegeneration. J. Physiol..

[71] André P., Samieri C., Buisson C., Dartigues J.-F., Helmer C., Laugerette F., Féart C. (2019). Lipopolysaccharide-binding protein, soluble cd14, and the long-term risk of alzheimer’s disease: A nested case-control pilot study of older community dwellers from the three-city cohort. J. Alzheimer’s Dis..

[72] Zamudio F., Loon A.R., Smeltzer S., Benyamine K., Navalpur Shanmugam N.K., Stewart N.J., Lee D.C., Nash K., Selenica M.-L.B. (2020). Tdp-43 mediated blood-brain barrier permeability and leukocyte infiltration promote neurodegeneration in a low-grade systemic inflammation mouse model. J. Neuroinflamm..

[73] Miklossy J. (2015). Historic evidence to support a causal relationship between spirochetal infections and alzheimer’s disease. Front. Aging Neurosci..

[74] Keshavarzian A., Engen P., Bonvegna S., Cilia R. (2020). The gut microbiome in parkinson’s disease: A culprit or a bystander?. Prog. Brain Res..

[75] Svoboda E. (2020). Could the gut microbiome be linked to autism?. Nature.

[76] Zhou Y., Wang Y., Quan M., Zhao H., Jia J. (2021). Gut microbiota changes and their correlation with cognitive and neuropsychiatric symptoms in alzheimer’s disease. J. Alzheimer’s Dis..

[77] Matheson J.-A.T., Holsinger R.D. (2023). The role of fecal microbiota transplantation in the treatment of neurodegenerative diseases: A review. Int. J. Mol. Sci..

[78] Fu J., Li J., Sun Y., Liu S., Song F., Liu Z. (2023). In-depth investigation of the mechanisms of schisandra chinensis polysaccharide mitigating alzheimer’s disease rat via gut microbiota and feces metabolomics. Int. J. Biol. Macromol..

[79] Xu Q.-Q., Su Z.-R., Yang W., Zhong M., Xian Y.-F., Lin Z.-X. (2023). Patchouli alcohol attenuates the cognitive deficits in a transgenic mouse model of alzheimer’s disease via modulating neuropathology and gut microbiota through suppressing c/ebpβ/aep pathway. J. Neuroinflamm..

[80] Wasén C., Simonsen E., Ekwudo M.N., Profant M.R., Cox L.M. (2022). The emerging role of the microbiome in alzheimer’s disease. Int. Rev. Neurobiol..

[81] Porsteinsson A.P., Isaacson R.S., Knox S., Sabbagh M.N., Rubino I. (2021). Diagnosis of early alzheimer’s disease: Clinical practice in 2021. J. Prev. Alzheimer’s Dis..

[82] Gauthreaux K., Bonnett T.A., Besser L.M., Brenowitz W.D., Teylan M., Mock C., Chen Y.C., Chan K.C.G., Keene C.D., Zhou X.H. (2020). Concordance of clinical alzheimer diagnosis and neuropathological features at autopsy. J. Neuropathol. Exp. Neurol..

[83] Liss J.L., Seleri Assunção S., Cummings J., Atri A., Geldmacher D.S., Candela S.F., Devanand D.P., Fillit H.M., Susman J., Mintzer J. (2021). Practical recommendations for timely, accurate diagnosis of symptomatic alzheimer’s disease (mci and dementia) in primary care: A review and synthesis. J. Intern. Med..

[84] Pais M., Martinez L., Ribeiro O., Loureiro J., Fernandez R., Valiengo L., Canineu P., Stella F., Talib L., Radanovic M. (2020). Early diagnosis and treatment of alzheimer’s disease: New definitions and challenges. Rev. Bras. Psiquiatr..

[85] Gebert L.F.R., MacRae I.J. (2019). Regulation of microrna function in animals. Nat. Rev. Mol. Cell Biol..

[86] Rani S. (2017). Microrna Profiling.

[87] Lee Y., Jeon K., Lee J.-T., Kim S., Kim V.N. (2002). Microrna maturation: Stepwise processing and subcellular localization. EMBO J..

[88] Yi R., Qin Y., Macara I.G., Cullen B.R. (2003). Exportin-5 mediates the nuclear export of pre-micrornas and short hairpin rnas. Genes Dev..

[89] Lund E., Guttinger S., Calado A., Dahlberg J.E., Kutay U. (2004). Nuclear export of microrna precursors. Science.

[90] Bohnsack M.T., Czaplinski K., Görlich D. (2004). Exportin 5 is a rangtp-dependent dsrna-binding protein that mediates nuclear export of pre-mirnas. RNA.

[91] Diederichs S., Haber D.A. (2007). Dual role for argonautes in microrna processing and posttranscriptional regulation of microrna expression. Cell.

[92] Raisch J., Darfeuille-Michaud A., Nguyen H.T.T. (2013). Role of micrornas in the immune system, inflammation and cancer. World J. Gastroenterol..

[93] Valencia-Sanchez M.A., Liu J., Hannon G.J., Parker R. (2006). Control of translation and mrna degradation by mirnas and sirnas. Genes Dev..

[94] O’Brien J., Hayder H., Zayed Y., Peng C. (2018). Overview of microrna biogenesis, mechanisms of actions, and circulation. Front. Endocrinol..

[95] Dexheimer P.J., Cochella L. (2020). Micrornas: From mechanism to organism. Front. Cell Dev. Biol..

[96] Ma Q., Zhang L., Pearce W.J. (2019). Micrornas in brain development and cerebrovascular pathophysiology. Am. J. Physiol. Cell Physiol..

[97] Saliminejad K., Khorram Khorshid H.R., Soleymani Fard S., Ghaffari S.H. (2019). An overview of micrornas: Biology, functions, therapeutics, and analysis methods. J. Cell. Physiol..

[98] Cho K.H.T., Xu B., Blenkiron C., Fraser M. (2019). Emerging roles of mirnas in brain development and perinatal brain injury. Front. Physiol..

[99] Angelucci F., Cechova K., Valis M., Kuca K., Zhang B., Hort J. (2019). Micrornas in alzheimer’s disease: Diagnostic markers or therapeutic agents?. Front. Pharmacol..

[100] Silvestro S., Bramanti P., Mazzon E. (2019). Role of mirnas in alzheimer’s disease and possible fields of application. Int. J. Mol. Sci..

[101] Roy B., Lee E., Li T., Rampersaud M. (2022). Role of mirnas in neurodegeneration: From disease cause to tools of biomarker discovery and therapeutics. Genes.

[102] Walgrave H., Zhou L., De Strooper B., Salta E. (2021). The promise of microrna-based therapies in alzheimer’s disease: Challenges and perspectives. Mol. Neurodegener..

[103] Lukiw W.J., Andreeva T.V., Grigorenko A.P., Rogaev E.I. (2012). Studying micro rna function and dysfunction in alzheimer’s disease. Front. Genet..

[104] Schratt G. (2009). Micrornas at the synapse. Nat. Rev. Neurosci..

[105] Zhang T.-N., Li D., Xia J., Wu Q.-J., Wen R., Yang N., Liu C.-F. (2017). Non-coding rna: A potential biomarker and therapeutic target for sepsis. Oncotarget.

[106] Winkle M., El-Daly S.M., Fabbri M., Calin G.A. (2021). Noncoding rna Therapeutics—Challenges and potential solutions. Nat. Rev. Drug Discov..

[107] Loganathan T., Doss C G.P. (2023). Non-coding rnas in human health and disease: Potential function as biomarkers and therapeutic targets. Funct. Integr. Genom..

[108] Fu X.-D. (2014). Non-coding rna: A new frontier in regulatory biology. Natl. Sci. Rev..

[109] Ylera F., Lurz R., Erdmann V.A., Fürste J.P. (2002). Selection of rna aptamers to the alzheimer’s disease amyloid peptide. Biochem. Biophys. Res. Commun..

[110] Grabowska-Pyrzewicz W., Want A., Leszek J., Wojda U. (2021). Antisense oligonucleotides for alzheimer’s disease therapy: From the mrna to mirna paradigm. EBioMedicine.

[111] Gyorgy B., Ingelsson M., Loov C., Takeda S., Lannfelt L., Hyman B.T., Breakefield X.O. (2016). 567. Crispr-cas9 mediated gene editing in a monogenic form of alzheimer’s disease. Mol. Ther..

[112] Pasquinelli A.E. (2012). Micrornas and their targets: Recognition, regulation and an emerging reciprocal relationship. Nat. Rev. Genet..

[113] Bonneau E., Neveu B., Kostantin E., Tsongalis G.J., De Guire V. (2019). How close are mirnas from clinical practice? A perspective on the diagnostic and therapeutic market. Ejifcc.

[114] Kyriakis J.M., Avruch J. (2012). Mammalian mapk signal transduction pathways activated by stress and inflammation: A 10-year update. Physiol. Rev..

[115] Peti W., Page R. (2013). Molecular basis of map kinase regulation. Protein Sci..

[116] Ryu H.-H., Lee Y.-S. (2016). Cell type-specific roles of ras-mapk signaling in learning and memory: Implications in neurodevelopmental disorders. Neurobiol. Learn. Mem..

[117] Pimienta G., Pascual J. (2007). Canonical and alternative mapk signaling. Cell Cycle.

[118] Turjanski A., Vaque J., Gutkind J. (2007). Map kinases and the control of nuclear events. Oncogene.

[119] Zhang Y., Dong C. (2007). Regulatory mechanisms of mitogen-activated kinase signaling. Cell. Mol. Life Sci..

[120] Knight T., Irving J.A.E. (2014). Ras/raf/mek/erk pathway activation in childhood acute lymphoblastic leukemia and its therapeutic targeting. Front. Oncol..

[121] Shaul Y.D., Seger R. (2007). The mek/erk cascade: From signaling specificity to diverse functions. Biochim. Biophys. Acta (BBA)-Mol. Cell Res..

[122] Johnson G.L., Lapadat R. (2002). Mitogen-activated protein kinase pathways mediated by erk, jnk, and p38 protein kinases. Science.

[123] Yang Y., Kim S.C., Yu T., Yi Y.-S., Rhee M.H., Sung G.-H., Yoo B.C., Cho J.Y. (2014). Functional roles of p38 mitogen-activated protein kinase in macrophage-mediated inflammatory responses. Mediat. Inflamm..

[124] Cuenda A., Rousseau S. (2007). P38 map-kinases pathway regulation, function and role in human diseases. Biochim. Biophys. Acta (BBA)-Mol. Cell Res..

[125] Cuadrado A., Nebreda A.R. (2010). Mechanisms and functions of p38 mapk signalling. Biochem. J..

[126] Zhang W., Liu H.T. (2002). Mapk signal pathways in the regulation of cell proliferation in mammalian cells. Cell Res..

[127] Sabio G., Davis R.J. (2014). Tnf and map kinase signalling pathways. Semin. Immunol..

[128] Yue J., López J.M. (2020). Understanding mapk signaling pathways in apoptosis. Int. J. Mol. Sci..

[129] Stramucci L., Pranteda A., Stravato A., Amoreo C.A., Pennetti A., Diodoro M.G., Bartolazzi A., Milella M., Bossi G. (2019). Mkk3 sustains cell proliferation and survival through p38delta mapk activation in colorectal cancer. Cell Death Dis..

[130] Bonni A., Brunet A., West A.E., Datta S.R., Takasu M.A., Greenberg M.E. (1999). Cell survival promoted by the ras-mapk signaling pathway by transcription-dependent and -independent mechanisms. Science.

[131] Fyffe-Maricich S.L., Karlo J.C., Landreth G.E., Miller R.H. (2011). The erk2 mitogen-activated protein kinase regulates the timing of oligodendrocyte differentiation. J. Neurosci..

[132] Imamura O., Pagès G., Pouysségur J., Endo S., Takishima K. (2010). Erk1 and erk2 are required for radial glial maintenance and cortical lamination. Genes Cells.

[133] Samuels I.S., Karlo J.C., Faruzzi A.N., Pickering K., Herrup K., Sweatt J.D., Saitta S.C., Landreth G.E. (2008). Deletion of erk2 mitogen-activated protein kinase identifies its key roles in cortical neurogenesis and cognitive function. J. Neurosci..

[134] Kim E.K., Choi E.-J. (2010). Pathological roles of mapk signaling pathways in human diseases. Biochim. Biophys. Acta (BBA)-Mol. Basis Dis..

[135] Kheiri G., Dolatshahi M., Rahmani F., Rezaei N. (2019). Role of p38/mapks in alzheimer’s disease: Implications for amyloid beta toxicity targeted therapy. Rev. Neurosci..

[136] Son Y., Cheong Y.K., Kim N.H., Chung H.T., Kang D.G., Pae H.O. (2011). Mitogen-activated protein kinases and reactive oxygen species: How can ros activate mapk pathways?. J. Signal Transduct..

[137] Lee J.K., Kim N.-J. (2017). Recent advances in the inhibition of p38 mapk as a potential strategy for the treatment of alzheimer’s disease. Molecules.

[138] Li T., Shi H., Zhao Y. (2018). Phosphorylation of microtubule-associated protein tau by mitogen-activated protein kinase in alzheimer’s disease. IOP Conf. Ser. Mater. Sci. Eng..

[139] Bazrgar M., Khodabakhsh P., Mohagheghi F., Prudencio M., Ahmadiani A. (2020). Brain micrornas dysregulation: Implication for missplicing and abnormal post-translational modifications of tau protein in alzheimer’s disease and related tauopathies. Pharmacol. Res..

[140] Wei W., Norton D.D., Wang X., Kusiak J.W. (2002). Abeta 17-42 in alzheimer’s disease activates jnk and caspase-8 leading to neuronal apoptosis. Brain.

[141] Xu H., Liu X., Li W., Xi Y., Su P., Meng B., Shao X., Tang B., Yang Q., Mao Z. (2021). P38 mapk-mediated loss of nuclear rnase iii enzyme drosha underlies amyloid beta-induced neuronal stress in alzheimer’s disease. Aging Cell.

[142] Mendell J.T., Olson E.N. (2012). Micrornas in stress signaling and human disease. Cell.

[143] Zeng L., Jiang H., Ashraf G.M., Liu J., Wang L., Zhao K., Liu M., Li Z., Liu R. (2022). Implications of mir-148a-3p/p35/pten signaling in tau hyperphosphorylation and autoregulatory feedforward of akt/creb in alzheimer’s disease. Mol. Ther. Nucleic Acids.

[144] Zhu X., Rottkamp C.A., Boux H., Takeda A., Perry G., Smith M.A. (2000). Activation of p38 kinase links tau phosphorylation, oxidative stress, and cell cycle-related events in alzheimer disease. J. Neuropathol. Exp. Neurol..

[145] Lok K., Zhao H., Shen H., Wang Z., Gao X., Zhao W., Yin M. (2013). Characterization of the app/ps1 mouse model of alzheimer’s disease in senescence accelerated background. Neurosci. Lett..

[146] Tan L., Yu J.T., Tan M.S., Liu Q.Y., Wang H.F., Zhang W., Jiang T., Tan L. (2014). Genome-wide serum microrna expression profiling identifies serum biomarkers for alzheimer’s disease. J. Alzheimer’s Dis..

[147] Arora T., Prashar V., Singh R., Barwal T.S., Changotra H., Sharma A., Parkash J. (2022). Dysregulated mirnas in progression and pathogenesis of alzheimer’s disease. Mol. Neurobiol..

[148] Fu Y., Hu X., Zheng C., Sun G., Xu J., Luo S., Cao P. (2019). Intrahippocampal mir-342-3p inhibition reduces β-amyloid plaques and ameliorates learning and memory in alzheimer’s disease. Metab. Brain Dis..

[149] Sterniczuk R., Antle M.C., Laferla F.M., Dyck R.H. (2010). Characterization of the 3xtg-ad mouse model of alzheimer’s disease: Part 2. Behavioral and cognitive changes. Brain Res..

[150] Kirouac L., Rajic A.J., Cribbs D.H., Padmanabhan J. (2017). Activation of ras-erk signaling and gsk-3 by amyloid precursor protein and amyloid beta facilitates neurodegeneration in alzheimer’s disease. eNeuro.

[151] Yarza R., Vela S., Solas M., Ramirez M.J. (2015). C-jun n-terminal kinase (jnk) signaling as a therapeutic target for alzheimer’s disease. Front. Pharmacol..

[152] Lukiw W.J. (2007). Micro-rna speciation in fetal, adult and alzheimer’s disease hippocampus. Neuroreport.

[153] Sethi P., Lukiw W.J. (2009). Micro-rna abundance and stability in human brain: Specific alterations in alzheimer’s disease temporal lobe neocortex. Neurosci. Lett..

[154] Liu S., Fan M., Zheng Q., Hao S., Yang L., Xia Q., Qi C., Ge J. (2022). Micrornas in alzheimer’s disease: Potential diagnostic markers and therapeutic targets. Biomed. Pharmacother. Biomed. Pharmacother..

[155] Jin Y., Tu Q., Liu M. (2018). Microrna-125b regulates alzheimer’s disease through sphk1 regulation. Mol. Med. Rep..

[156] Banzhaf-Strathmann J., Benito E., May S., Arzberger T., Tahirovic S., Kretzschmar H., Fischer A., Edbauer D. (2014). Microrna-125b induces tau hyperphosphorylation and cognitive deficits in alzheimer’s disease. EMBO J..

[157] Ma Q.L., Harris-White M.E., Ubeda O.J., Simmons M., Beech W., Lim G.P., Teter B., Frautschy S.A., Cole G.M. (2007). Evidence of abeta- and transgene-dependent defects in erk-creb signaling in alzheimer’s models. J. Neurochem..

[158] Hernandez-Rapp J., Smith P.Y., Filali M., Goupil C., Planel E., Magill S.T., Goodman R.H., Hebert S.S. (2015). Memory formation and retention are affected in adult mir-132/212 knockout mice. Behav. Brain Res..

[159] Wanet A., Tacheny A., Arnould T., Renard P. (2012). Mir-212/132 expression and functions: Within and beyond the neuronal compartment. Nucleic Acids Res..

[160] Cogswell J.P., Ward J., Taylor I.A., Waters M., Shi Y., Cannon B., Kelnar K., Kemppainen J., Brown D., Chen C. (2008). Identification of mirna changes in alzheimer’s disease brain and csf yields putative biomarkers and insights into disease pathways. J. Alzheimer’s Dis..

[161] Lau P., Bossers K., Janky R., Salta E., Frigerio C.S., Barbash S., Rothman R., Sierksma A.S., Thathiah A., Greenberg D. (2013). Alteration of the microrna network during the progression of alzheimer’s disease. EMBO Mol. Med..

[162] Smith P.Y., Hernandez-Rapp J., Jolivette F., Lecours C., Bisht K., Goupil C., Dorval V., Parsi S., Morin F., Planel E. (2015). Mir-132/212 deficiency impairs tau metabolism and promotes pathological aggregation in vivo. Hum. Mol. Genet..

[163] Hebert S.S., Wang W.X., Zhu Q., Nelson P.T. (2013). A study of small rnas from cerebral neocortex of pathology-verified alzheimer’s disease, dementia with lewy bodies, hippocampal sclerosis, frontotemporal lobar dementia, and non-demented human controls. J. Alzheimer’s Dis..

[164] Hernandez-Rapp J., Rainone S., Goupil C., Dorval V., Smith P.Y., Saint-Pierre M., Vallée M., Planel E., Droit A., Calon F. (2016). Microrna-132/212 deficiency enhances aβ production and senile plaque deposition in alzheimer’s disease triple transgenic mice. Sci. Rep..

[165] Salta E., Sierksma A., Vanden Eynden E., De Strooper B. (2016). Mir-132 loss de-represses itpkb and aggravates amyloid and tau pathology in alzheimer’s brain. EMBO Mol. Med..

[166] Nagaraj S., Want A., Laskowska-Kaszub K., Fesiuk A., Vaz S., Logarinho E., Wojda U. (2021). Candidate alzheimer’s disease biomarker mir-483-5p lowers tau phosphorylation by direct erk1/2 repression. Int. J. Mol. Sci..

[167] Pietersen C.Y., Mauney S.A., Kim S.S., Lim M.P., Rooney R.J., Goldstein J.M., Petryshen T.L., Seidman L.J., Shenton M.E., McCarley R.W. (2014). Molecular profiles of pyramidal neurons in the superior temporal cortex in schizophrenia. J. Neurogenet..

[168] Kim W., Noh H., Lee Y., Jeon J., Shanmugavadivu A., McPhie D.L., Kim K.S., Cohen B.M., Seo H., Sonntag K.C. (2016). Mir-126 regulates growth factor activities and vulnerability to toxic insult in neurons. Mol. Neurobiol..

[169] Bluthgen N., van Bentum M., Merz B., Kuhl D., Hermey G. (2017). Profiling the mapk/erk dependent and independent activity regulated transcriptional programs in the murine hippocampus in vivo. Sci. Rep..

[170] Zou J., Lei T., Guo P., Yu J., Xu Q., Luo Y., Ke R., Huang D. (2019). Mechanisms shaping the role of erk1/2 in cellular senescence (review). Mol. Med. Rep..

[171] Schulte-Herbruggen O., Jockers-Scherubl M.C., Hellweg R. (2008). Neurotrophins: From pathophysiology to treatment in alzheimer’s disease. Curr. Alzheimer Res..

[172] Xu S., Zhang R., Niu J., Cui D., Xie B., Zhang B., Lu K., Yu W., Wang X., Zhang Q. (2012). Oxidative stress mediated-alterations of the microrna expression profile in mouse hippocampal neurons. Int. J. Mol. Sci..

[173] Zhang R., Zhang Q., Niu J., Lu K., Xie B., Cui D., Xu S. (2014). Screening of micrornas associated with alzheimer’s disease using oxidative stress cell model and different strains of senescence accelerated mice. J. Neurol. Sci..

[174] Deng Y., Zhang J., Sun X., Ma G., Luo G., Miao Z., Song L. (2020). Mir-132 improves the cognitive function of rats with alzheimer’s disease by inhibiting the mapk1 signal pathway. Exp. Ther. Med..

[175] Shi Z., Zhang K., Zhou H., Jiang L., Xie B., Wang R., Xia W., Yin Y., Gao Z., Cui D. (2020). Increased mir-34c mediates synaptic deficits by targeting synaptotagmin 1 through ros-jnk-p53 pathway in alzheimer’s disease. Aging Cell.

[176] Müller M., Kuiperij H.B., Claassen J.A., Küsters B., Verbeek M.M. (2014). Micrornas in alzheimer’s disease: Differential expression in hippocampus and cell-free cerebrospinal fluid. Neurobiol. Aging.

[177] Beard J.A., Tenga A., Hills J., Hoyer J.D., Cherian M.T., Wang Y.D., Chen T. (2016). The orphan nuclear receptor nr4a2 is part of a p53-microrna-34 network. Sci. Rep..

[178] Cortez M.A., Ivan C., Valdecanas D., Wang X., Peltier H.J., Ye Y., Araujo L., Carbone D.P., Shilo K., Giri D.K. (2016). Pdl1 regulation by p53 via mir-34. J. Natl. Cancer Inst..

[179] Bhatnagar S., Chertkow H., Schipper H.M., Yuan Z., Shetty V., Jenkins S., Jones T., Wang E. (2014). Increased microrna-34c abundance in alzheimer’s disease circulating blood plasma. Front. Mol. Neurosci..

[180] Xie Y., Chen Y. (2016). Micrornas: Emerging targets regulating oxidative stress in the models of parkinson’s disease. Front. Neurosci..

[181] Fioravanti A., Giordano A., Dotta F., Pirtoli L. (2022). Crosstalk between microrna and oxidative stress in physiology and pathology 2.0. Int. J. Mol. Sci..

[182] Jadhav S.P., Kamath S.P., Choolani M., Lu J., Dheen S.T. (2014). Microrna-200b modulates microglia-mediated neuroinflammation via the cjun/mapk pathway. J. Neurochem..

[183] Liang X., Wang L., Wang M., Liu Z., Liu X., Zhang B., Liu E., Li G. (2020). Microrna-124 inhibits macrophage cell apoptosis via targeting p38/mapk signaling pathway in atherosclerosis development. Aging.

[184] Shang L., Peng T., Chen X., Yan Z., Wang J., Gao X., Chang C. (2022). Mir-590-5p overexpression alleviates β-amyloid-induced neuron damage via targeting pellino-1. Anal. Cell. Pathol..

[185] Freeman R.S., Estus S., Johnson E.M. (1994). Analysis of cell cycle-related gene expression in postmitotic neurons: Selective induction of cyclin d1 during programmed cell death. Neuron.

[186] Yang Y., Herrup K. (2007). Cell division in the cns: Protective response or lethal event in post-mitotic neurons?. Biochim. Biophys. Acta.

[187] Webber K.M., Raina A.K., Marlatt M.W., Zhu X., Prat M.I., Morelli L., Casadesus G., Perry G., Smith M.A. (2005). The cell cycle in alzheimer disease: A unique target for neuropharmacology. Mech. Ageing Dev..

[188] Park D.S., Obeidat A., Giovanni A., Greene L.A. (2000). Cell cycle regulators in neuronal death evoked by excitotoxic stress: Implications for neurodegeneration and its treatment. Neurobiol. Aging.

[189] Modi P.K., Komaravelli N., Singh N., Sharma P. (2012). Interplay between mek-erk signaling, cyclin d1, and cyclin-dependent kinase 5 regulates cell cycle reentry and apoptosis of neurons. Mol. Biol. Cell.

[190] Modi P.K., Jaiswal S., Sharma P. (2016). Regulation of neuronal cell cycle and apoptosis by microrna 34a. Mol. Cell. Biol..

[191] Yang Y., Mufson E.J., Herrup K. (2003). Neuronal cell death is preceded by cell cycle events at all stages of alzheimer’s disease. J. Neurosci..

[192] He B., Chen W., Zeng J., Tong W., Zheng P. (2020). Microrna-326 decreases tau phosphorylation and neuron apoptosis through inhibition of the jnk signaling pathway by targeting vav1 in alzheimer’s disease. J. Cell. Physiol..

[193] Cardoso A.L., Guedes J.R., Pereira de Almeida L., Pedroso de Lima M.C. (2012). Mir-155 modulates microglia-mediated immune response by down-regulating socs-1 and promoting cytokine and nitric oxide production. Immunology.

[194] Zingale V.D., Gugliandolo A., Mazzon E. (2021). Mir-155: An important regulator of neuroinflammation. Int. J. Mol. Sci..

[195] Guedes J.R., Custódia C.M., Silva R.J., de Almeida L.P., Pedroso de Lima M.C., Cardoso A.L. (2014). Early mir-155 upregulation contributes to neuroinflammation in alzheimer’s disease triple transgenic mouse model. Hum. Mol. Genet..

[196] Colonna M., Butovsky O. (2017). Microglia function in the central nervous system during health and neurodegeneration. Annu. Rev. Immunol..

[197] Wan W., Liu G., Li X., Liu Y., Wang Y., Pan H., Hu J. (2021). Mir-191-5p alleviates microglial cell injury by targeting map3k12 (mitogen-activated protein kinase kinase kinase 12) to inhibit the mapk (mitogen-activated protein kinase) signaling pathway in alzheimer’s disease. Bioengineered.

